# Checklist of vascular plant species in Huangshui River Basin of Qinghai Province, China

**DOI:** 10.3897/BDJ.12.e123002

**Published:** 2024-05-22

**Authors:** Yuqi Ma, Chunjing Wang, Chuping Wu, Shanfeng Huang, Zhiwen Gao, Zhi Chen, Feihai Yu, Chunhui Zhang, Jizhong Wan

**Affiliations:** 1 College of Life Science, Qinghai Normal University, Xining, China College of Life Science, Qinghai Normal University Xining China; 2 State Key Laboratory of Plateau Ecology and Agriculture, Qinghai University, Xining, China State Key Laboratory of Plateau Ecology and Agriculture, Qinghai University Xining China; 3 Zhejiang Academy of Forestry, Hangzhou, China Zhejiang Academy of Forestry Hangzhou China; 4 Climate Bridge Ltd (Shanghai), Shanghai, China Climate Bridge Ltd (Shanghai) Shanghai China; 5 Institute of Wetland Ecology & Clone Ecology/Zhejiang Provincial Key Laboratory of Evolutionary Ecology and Conservation, Taizhou University, Taizhou, Zhejiang, China Institute of Wetland Ecology & Clone Ecology/Zhejiang Provincial Key Laboratory of Evolutionary Ecology and Conservation, Taizhou University Taizhou, Zhejiang China

**Keywords:** Alpine region, checklist, China, Huangshui River Basin, Qinghai Province, vascular plants

## Abstract

**Background:**

The Huangshui River Basin is one of the most important water sources in the Qinghai Province and is of great importance for ecological protection measures, agricultural irrigation and tourism. Based on previous studies and fieldwork related to plant species in China, this study presents comprehensive data on vascular plants distributed in the Huangshui River Basin of Qinghai Province.

Ethical Compliance: All procedures performed in studies involving human participants were in accordance with the ethical standards of the institutional and/or national research committee and with the 1964 Helsinki Declaration and its later amendments or comparable ethical standards.

Data Access Statement: Research data supporting this publication are available from the repository at located at https://www.scidb.cn/en/anonymous/QUpuZVEz.

Conflict of Interest declaration: The authors declare that they have NO affiliations with or involvement in any organisation or entity with any financial interest in the subject matter or materials discussed in this manuscript.

**New information:**

The checklist of plants includes ferns, gymnosperms and angiosperms, covering three phyla, five classes, 49 orders, 139 families, 709 genera and 2,382 species. It includes numerous Asteraceae, Gramineae, Rosaceae and Fabaceae along with statistical data on the number of species distributed in different regions. The dataset presented in this article provides important background information on vascular plants in the Huangshui River Basin and, therefore, plays a crucial role in the protection and management of plant resources in this region.

## Introduction

The Huangshui River is the largest tributary of the upper reaches of the Yellow River and is located in the transitional zone between the Loess Plateau and the Qinghai-Tibet Plateau, with a drainage basin area accounting for 2.3% of the land area of Qinghai. The Huangshui River Basin is an important component of the ecosystem of the Qinghai Tibet Plateau with rich biodiversity (Wang et al. 2023, Hao et al. 2023). It contains mountains, wetlands, lakes and rivers that provide habitats for numerous species. The wetlands in the Huangshui River Basin have important water source conservation and biodiversity functions, with marsh wetlands and river wetlands being the main types of wetlands (Huang et al. 2023).

Plant diversity has important ecological significance in ecosystems and higher plant diversity can enhance ecosystem stability (Pohl et al. 2009, Mazzochini et al. 2019). Plant diversity also plays an important role in the restoration of degraded ecosystems and high diversity promotes rapid restoration and reconstruction of ecosystems after human-induced damage or natural disasters (Standish et al. 2021, Liu et al. 2021).

As a result of comprehensive investigations of plant resources in the Huangshui River Basin, species are constantly being updated. In addition to the original records of some vascular plant taxa in the Huangshui River Basin that were merged after the classification revision, more information needs to be updated. This article is based on nine books on plant species in Qinghai Province. In addition, we conducted two field investigations on vascular plant communities in Xining City, Haidong City, Haibei Tibetan Autonomous Prefecture and the Huangshui River Basin from the summers of 2021 and 2022, complemented by an extensive literature search and verification (Wang et al. 2023). We used the latest classification systems for various groups to revise and update the catalogue of vascular plants in the Huangshui River Basin and recorded information on species, sub-taxonomic groups and distribution areas. Based on the above information, we generated the latest checklist for wild vascular plants in the Huangshui River Basin.

This dataset can help researchers and environmental organisations to understand the plant diversity status of the Huangshui River Basin. By gaining insight into the types and distribution of plants, we can develop corresponding protection measures to prevent the extinction of endangered species and protect overall species diversity. This checklist facilitates the development of research and monitoring strategies and plays a crucial role in the protection and management of plant resources in the Huangshui River Basin.

## Sampling methods

### Sampling description

To generate the checklist, we used the following publications: "Wild Medicinal Plants in Qinghai" (Lu and Zhang 2012), "Atlas of Vascular Plants in Hainan Tibetan Autonomous Prefecture, Qinghai Province" (Volumes 1 and 2) (Zhou 2020), "Flora of Qinghai " (Volumes 1–4) (Liu 1997), "Atlas of Main Grassland Types and Common Plants in Qinghai" (Hou and Sun 2012) and "List of Seed Plants in Qinghai" (Gou 1990). In addition, in the summer seasons of 2021 and 2022, we conducted two field surveys to investigate the communities of herbaceous plant species in Xining City, Haidong City and Haiyan County of Haibei Tibetan Autonomous Prefecture, sampling a total of 455 quadrats comprising 4,113 records and 277 plant species (55 families and 172 genera). Based on this information, we calculated the diversity indices (species richness, Shannon-Wiener index, Pielou index and Simpson index) of the plant communities. These data were integrated into the aforementioned data to generate the final checklist. In addition, we used the iPlant (http://www.iplant.cn) and the Chinese Field Herbarium (http://www.cfh.ac.cn). These two websites are plant intelligence information systems and biodiversity information platforms built by the Institute of Botany, Chinese Academy of Sciences. iPlant focuses on the field of plant science and provides the latest research results in plant science. Chinese Field Herbarium can provide services such as species information enquiry, photo storage and species identification.

### Quality control

The dataset consisted of 11 columns and could be divided into four parts: species information, geographic distribution information, data sources and national protection level. There were seven columns of species information, including Latin names for species, phyla, classes, orders, families, genera and nomenclature. The geographic distribution information, data sources and national protection levels each occupy one column (Appendix 1).

### Step description

The dataset was organised as follows: (1) Deduplication of data. Based on the literature and field survey data, all data were integrated into the original checklist of vascular plants in the Huangshui River Basin, Qinghai Province and a table was generated. The information was checked using iPlant (http://www.iplant.cn), supplemented with the Chinese Field Herbarium (http://www.cfh.ac.cn). We combined the names of the orders, families, genera and species in the latest classification system, modified the Latin scientific names of the species in the table, removed duplicates and retained the unique order, family and genus information for relevance; (2) The classification system is updated. In this step, we redefined and systematically arranged the three major categories of ferns, gymnosperms and angiosperms according to the corresponding systems. Ferns were classified according to the system of lycopods and ferns; gymnosperms were classified according to the system of gymnosperms; and angiosperms were classified according to the system of angiosperms; (3) Organisation of geographic distribution. Based on the literature mentioned above, we have improved the geographical distribution of the various species. We also retrieved specimen records using iPlant (http://www.iplant.cn) and the Chinese Field Herbarium (http://www.cfh.ac.cn); (4) Indication of the national protection level. According to the “List of National Key Protected Wild Plants in China” released by the State Forestry and Grassland Administration, the species on the checklist were labelled with national protection levels.

## Geographic coverage

### Description

The Huangshui River is a primary tributary of the upper Yellow River, originating from Haiyan County, Qinghai Province and is located in the transitional zone between the Qinghai-Tibet Plateau and the Loess Plateau. The total area of the Huangshui River Basin is about 16,100 km^2^. The climate is continental and the region shows significant terrain differences and a unique plant community. The Huangshui River Basin plays an important role in ensuring the biodiversity of Qinghai Lake and the surrounding wetlands, maintaining the water resource supply, supporting soil and water conservation and promoting local sustainable development.

### Coordinates

36° 19′ and 36° 53′ Latitude; 100°59′ and 102°48′ Longitude.

## Taxonomic coverage

### Description

General taxonomic coverage includes five classes, 49 orders, 139 families, 709 genera and 2,382 plant species. According to Flora of China, there are 301 families, 3,408 genera and 31,142 species of vascular plants in China. Vascular plants included in this checklist accounted for 8% of the total species of vascular plants in China.

## Usage licence

### Usage licence

Creative Commons Public Domain Waiver (CC-Zero)

## Data resources

### Data package title

Checklist of vascular plant species in Huangshui River Basin of Qinghai Province, China

### Resource link

https://www.scidb.cn/en/anonymous/QUpuZVEz

### Number of data sets

1

### Data set 1.

#### Data set name

Species details

#### Description

This checklist records the species, phyla, classes, orders, families, genera, nomenclature, geographic distribution information, data sources and national protection level. Each row represents a single species.

**Data set 1.** 

Column label	Column description
code	The identification number of plant species.
canonical_name	The full scientific name of the plant species.
author	Name of the person who named the plant.
phylum	The full scientific name of the plant phylum.
class	The full scientific name of the plant class.
order	The full scientific name of the plant order.
family	The full scientific name of the plant family.
genus	The full scientific name of the plant genus.
distribution	The distribution area of the plant.
literature	The literature for recording the plant.
national protection level	The national protection level of the plant.

## Additional information

### Analysis

**Figure 1.**
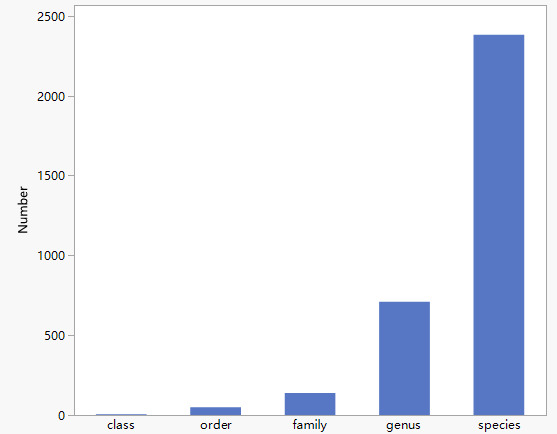
Numbers of plant classes, orders, families, genera and species in the Huangshui River Basin.

**Statistical analyses of families and genera**:

The dataset is presented in an Excel file. It includes 2,382 species belonging to five classes, 49 orders, 139 families and 709 genera (Fig. 1). It contains 27 species of pteridophytes (1 class, 2 orders, 9 families and 14 genera), 36 species of gymnosperms (3 classes, 5 orders, 5 families and 13 genera) and 2,319 species of angiosperms (1 class, 42 orders, 125 families and 682 genera). In addition, 192 variants and 56 subspecies were identified in the dataset.

The top 20 families with the highest numbers of species are Poaceae (63 genera and 232 species), Asteraceae (70 genera and 228 species), Rosaceae (29 genera and 181 species), Fabaceae (45 genera and 148 species), Ranunculaceae (21 genera and 111 species), Salicaceae (2 genera and 74 species), Brassicaceae (32 genera and 68 species), Apiaceae (24 genera and 58 species), Caryophyllaceae (15 genera and 55 species), Cyperaceae (8 genera and 53 species), Lamiaceae (23 genera and 51 species), Orobanchaceae (11 genera and 51 species), Gentianaceae (10 genera and 50 species), Orchidaceae (20 genera and 42 species), Polygonaceae (12 genera and 42 species), Amaranthaceae (20 genera and 39 species), Caprifoliaceae (8 genera and 37 species), Papaveraceae (9 genera and 37 species), Boraginaceae (11 genera and 30 species) and Asparagaceae (10 genera and 27 species).

**Figure 2.**
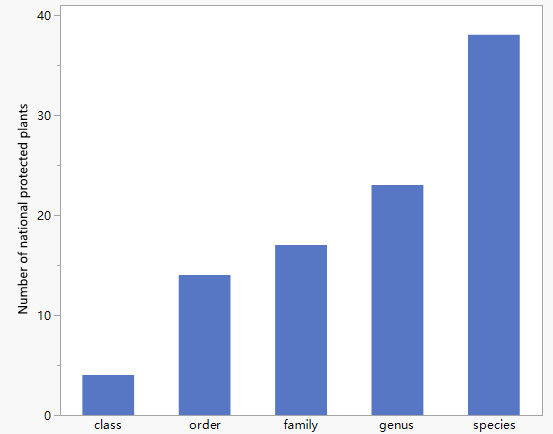
Number of national protected plants in the Huangshui River Basin.

**Statistical analyses of protected wild plants**:

According to the “List of National Key Protected Wild Plants in China” released by the State Forestry and Grassland Administration, the checklist contains 38 wild plants that are protected in China. They belong to four classes, 14 orders, 17 families and 23 genera (Fig. 2). Amongst these 38 species, three were in the first-level protection category (*Cycasrevoluta* (Thunb.), *Ginkgobiloba* (L.) and *Metasequoiaglyptostroboide*s (Hu & W. C. Cheng)) and 35 were in the second-level protection category.

**Figure 3.**
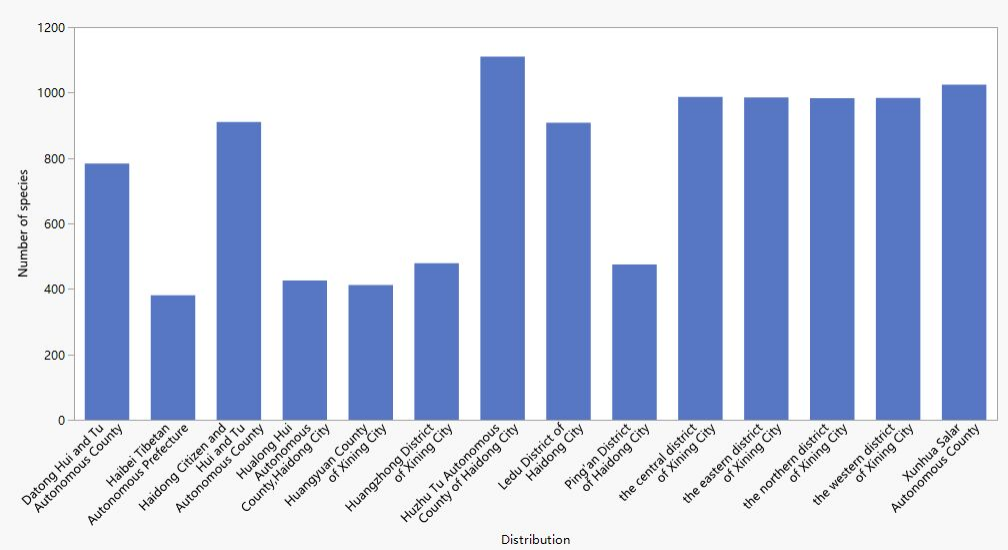
Numbers of plant species in the different regions of the Huangshui River Basin.

**Geographical distribution pattern**:

According to geographical distribution statistics, there are 985 species in the eastern district of Xining City, 987 species in the central district of Xining City, 984 species in the western district of Xining City, 983 species in the northern district of Xining City, 783 species in Datong Hui and Tu Autonomous County, 479 species in Huangzhong District of Xining City, 412 species in Huangyuan County of Xining City, 910 species in Haidong Citizen and Hui and Tu Autonomous County, 1,110 species in Huzhu Tu Autonomous County of Haidong City, 908 species in Ledu District of Haidong City, 475 species in Ping'an District of Haidong City, 426 species in Hualong Hui Autonomous County, Haidong City, 1,024 species in Xunhua Salar Autonomous County and 381 species in Haibei Tibetan Autonomous Prefecture (Fig. 3).

### Discussion

The Huangshui River Basin is an important ecological barrier in north-western China. Although it accounts for only 2.3% of the total area of Qinghai Province, it contains nearly 60% of the population, industry and agriculture in this Province. Based on remote sensing images, researchers evaluated the ecological environment of the Huangshui River Basin and concluded that its conditions were average (Zhao et al. 2018). However, as a result of global warming, the glaciers on the Qinghai-Tibet Plateau are retreating, which may affect the water resources in the Huangshui River Basin. Therefore, improving biodiversity can increase the stability of ecosystems in this region. For example, a higher plant diversity can reduce soil erosion, improve water quality and increase the sustainability of water sources. In this context, an updated checklist of the vascular plants in this region can improve our understanding of the distribution of local plant species, thereby facilitating the development of management and protection strategies. Based on our literature survey, there is no clear consensus amongst the scientific community regarding the types and distribution of vascular plants in the Huangshui River Basin, which necessitates the generation of a checklist. This checklist provides information about endangered or threatened plants in an area, thereby guiding government authorities in formulating relevant protection policies.

The Huangshui River Basin contains a variety of protected wild plant resources. Based on current knowledge, there are 38 species of national key-protected wild plants in 14 orders, 17 families and 22 genera. Many key protected wild plants in different countries are facing extinction, necessitating the implementation of suitable protection measures. Specialised wildlife and nature reserves can be established to create suitable habitats and protect the environment of endangered wild plants. In addition, it is necessary to strengthen habitat monitoring and population investigation of protected wild plants, providing the scientific basis for formulating protection measures. Education of the general public should be strengthened to raise awareness about the importance of national key protected wild plants.

Knowledge of the plant diversity of the Huangshui River Basin is crucial, as it improves our understanding of the current and future ecological environment and distribution of plant resources in this region. In this context, the checklist of vascular plant species provides a scientific basis for protecting the ecological environment, maintaining biodiversity and promoting sustainable development in this region. In addition, research on plant biodiversity can lead to the discovery of new plant resources and provide new research materials for fields such as agriculture, medicine and forestry.
